# Different Effects of Thiazolidinediones on In-Stent Restenosis and Target Lesion Revascularization after PCI: A Meta-Analysis of Randomized Controlled Trials

**DOI:** 10.1038/s41598-017-14873-0

**Published:** 2017-10-31

**Authors:** Xinbin Zhou, Shenjie Chen, Min Zhu, Junyi Hua, Jin Dai, Xiaoming Xu, Yuangang Qiu, Wei Mao

**Affiliations:** Department of Cardiology, First Affiliated Hospital of Zhejiang Chinese Medical University, Hangzhou310006, Zhejiang, China

## Abstract

In-stent restenosis (ISR) remains the leading problem encountered after percutaneous coronary intervention (PCI). Thiazolidinediones (TZDs) has been shown to be associated with reduced ISR and target lesion revascularization (TLR); however, the results are inconsistent, especially between rosiglitazone and pioglitazone. In this study, fourteen RCTs with a total of 1350 patients were finally included through a systematical literature search of Embase, Pubmed, the Cochrane Library, and ClinicalTrials.gov from inception to January 31, 2017. The follow-up duration of the included trials ranged from 6 months to 18 months. The results demonstrated that TZDs treatment is associated with significantly reduced risk of TLR (RR:0.45, 95%CI 0.30 to 0.67 for pioglitazone, RR:0.68, 95%CI 0.46 to 1.00 for rosiglitazone). Pioglitazone is associated with significantly reduced risks of ISR (RR:0.47, 95%CI 0.27 to 0.81), major adverse cardiac events (MACE) (RR:0.44, 95%CI 0.30 to 0.64) and neointimal area (SMD: −0.585, 95%CI −0.910 to −0.261). No significant relationship was observed between rosiglitazone and ISR (RR:0.91, 95%CI 0.39 to 2.12), MACE (RR:0.73, 95%CI 0.53 to 1.00) and neointimal area (SMD: −0.164, 95%CI −1.146 to 0.818). This meta-analysis demonstrated that TZDs treatment is associated with significant reduction in ISR, TLR and MACE for patients after PCI. Pioglitazone treatment seems to have more beneficial effects than rosiglitazone and no significantly increased cardiovascular risk was detected for both agents.

## Introduction

In-stent restenosis (ISR) remains a significant problem after percutaneous coronary intervention (PCI), both for the bare-metal stent (BMS) and drug-eluting stent (DES)^1,2^. Besides antiplatelet therapy, no additional drugs are routinely used to prevent ISR.

Thiazolidinediones (TZDs), as a class of agonists of the peroxisome proliferation-activated receptor-γ (PPAR-γ), have widely been used since 1990 as insulin sensitizers in the treatment of diabetes^3^.

This class of agonists can also modulate several biological processes to inhibit cellular proliferation and reduce inflammation after vascular injury^4–7^, adding to cardiovascular interest and promise. Rosiglitazone and pioglitazone currently are commercially available. Previous meta-analysis have demonstrated benefits of both TZDs and pioglitazone in prevention of ISR and target lesion revascularization (TLR)^8–12^. Few of these analyses are based on RCT data; the results of these studies are also inconsistent.

Because of the potential cardiac risk, as reported by several studies for rosiglitazone^13^, most studies focused only on pioglitazone; the different effects on ISR and TLR between rosiglitazone and pioglitazone have not been discussed and clearly demonstrated. However, reevaluation of the RECORD trial data demonstrated that rosiglitazone did not increase any risk of heart attack; therefore, its clinical restrictions eventually have been removed^14^.

This present meta-analysis, based on the updated information, was performed to examine further the role of TZDs in the prevention of ISR and TLR after PCI in diabetic and non-diabetic patients. Additionally, potential differences between rosiglitazone and pioglitazone were investigated.

## Materials and Methods

No ethical approval was required as all the data were acquired from previously published studies.

### Search Strategy and Selection Criteria

We systematically searched articles on effects of TZDs after PCI with Embase, Pubmed, the Cochrane Library, and ClinicalTrials.gov from inception to January 31, 2017. The following terms and variants thereof were used: “stent”, “restenosis”, “thiazolidinediones”, “rosiglitazone”, “pioglitazone.” Additionally, the references of the selected articles, relevant reviews and previous meta-analyses were manually searched for potentially relevant citations. Only RCTs in the English language with full article text were included.

Studies were required to meet the following criteria to be included in the research: (1) randomized controlled trial (RCT), (2) original data showing the effects of TZDs after PCI, (3) TZDs therapy compared with placebo, without TZDs, or other anti-diabetic therapy, (4) the outcomes of interest were reported, and (5) the length of follow-up was at least 6 months. RCTs concerned with troglitazone or without a full article were excluded.

### Data Collection and Quality Assessment

Two reviewers performed the data extraction and quality assessment independently, and disagreements were resolved by consensus. The following data were extracted: number of patients assigned to each group, participant characteristics, TZDs type, duration of follow-up, and outcomes of interest. The quality of the RCTs included was assessed with the Cochrane Collaboration tool^15^.

### Outcomes

The primary outcomes of interest were the number of patients with angiographic ISR by quantified coronary angiography (QCA) and the patients required to have TLR during follow-up. Secondary outcomes included major adverse cardiac events (MACE) and other QCA results including late lumen loss (LLL), minimum lumen diameter (MLD) and percentage stenosis (PS). The most frequently used intravascular ultrasound (IVUS) measurements, average in-stent neointimal area (neointimal volume/stent length) and neointimal index (neointimal volume/stent volume or neointimal area/stent area) were also analysed if IVUS procedure was performed.

### Statistical Analysis

STATA version 12.0 (STATA Corporation, TX, USA) was used to perform statistical analysis. Relative risk (RR) or standard mean difference (SMD) and their 95% confidence intervals (CIs) were calculated to demonstrate the overall result. Heterogeneity across studies was assessed with the chi-square test, and I^2^ > 50% was considered indicative of significant heterogeneity. The causes were investigated and a random effects model was applied when a significant heterogeneity was present; otherwise, a fixed effects model was used. Publication bias was analysed graphically with funnel plots and statistically with Egger’s and Begg’s tests. A two-sided P value of < 0.05 was considered to be statistically significant.

## Results

### Eligible Studies and Characteristics

**Figure 1:**
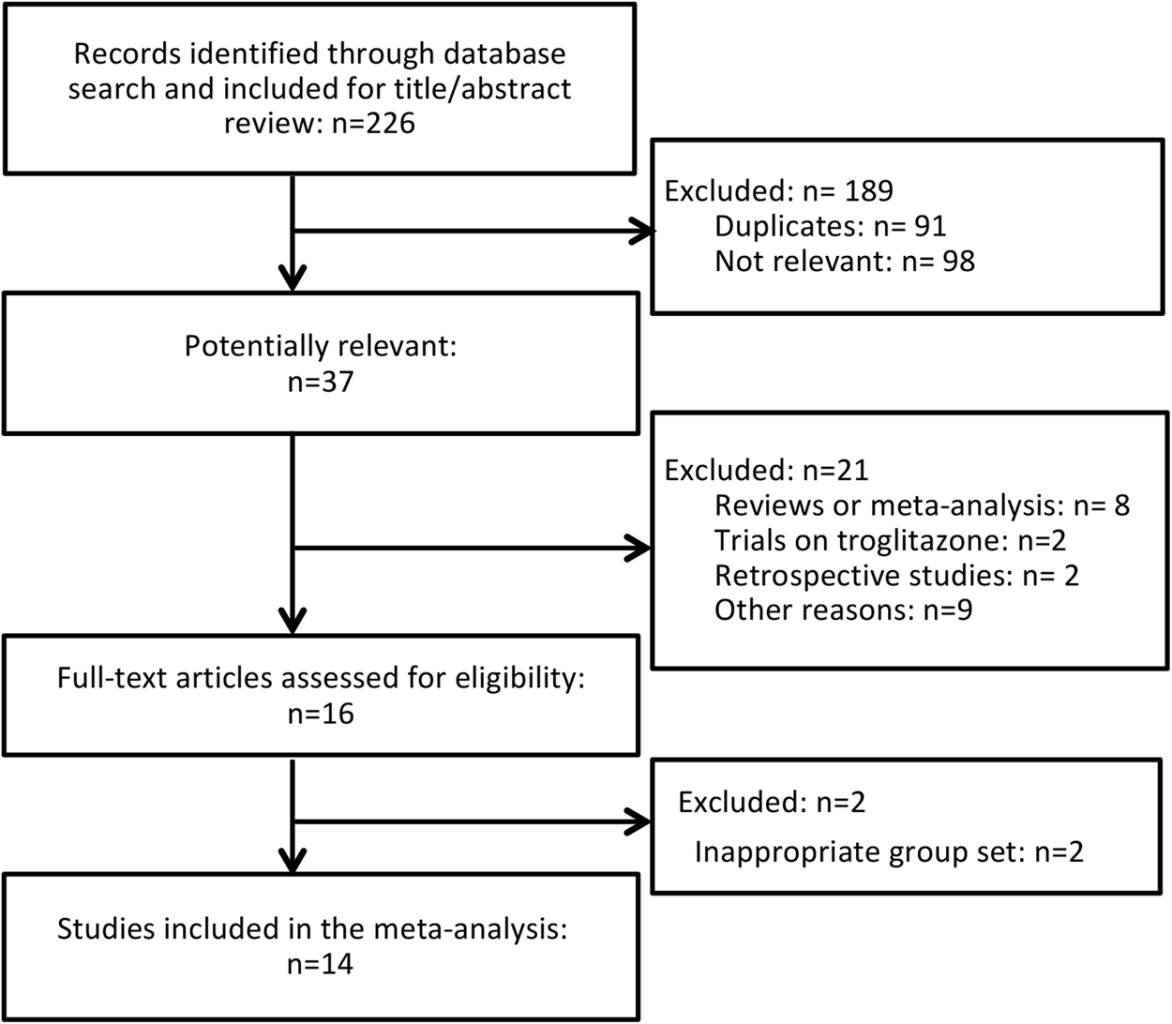
Flow chart of the systematic literature research.

A total of 226 studies were identified in the initial search, of which 37 studies were further assessed. Ultimately, 14 RCTs with a total of 1350 patients were included in the analysis, with follow-up ranging from 6 months to 18 months after intervention^16–29^. No additional studies were identified when we manually searched the references of the included articles and relevant reviews (Fig. 1). The baseline characteristics of the included studies are outlined in Table 1. Briefly, 8 trials were treated with pioglitazone^20–25,27,28^, and 6 trials were treated with rosiglitazone^16–19,26,29^.

**Table 1:** Baseline characteristics of the selected trials.

Study	Year	Study population	Mean age (TZDs/Cont), year	N (TZDs/Cont)	Interventions (TZDs type and dose)	Follow-up, months
Takagai *et al*.	2003	Type2 DM	64.0/65.0	23/21	Pioglitazone, 30 mg/d	6
Choi *et al*.	2004	Type2 DM	60.9/59.9	38/45	Rosiglitazone, 8 mg/d	6
Osman *et al*.	2004	Type2 DM	53.5/57.3	8/8	Rosiglitazone, 8 mg/d	6
Marx *et al*.	2005	Non-DM	63.4/60.8	29/31	Pioglitazone, 30 mg/d	6
Wang *et al*.	2005	Type2 DM	60.1/62.2	35/35	Rosiglitazone, 4 mg/d	6
Cao *et al*.	2006	Metabolic syndrome	60.6/59.5	152/145	Rosiglitazone, 4 mg/d	9
Nishio *et al*.	2006	Type2 DM	66.2/67.5	26/28	Pioglitazone, 30 mg/d	6
Katayama *et al*.	2007	Metabolic syndrome	60.1/61.3	16/16	Pioglitazone, 30 mg/d	6
Finn *et al*.	2009	Type2 DM	65.7/62.6	32/33	Rosiglitazone, 4 mg/d	8
Takagi *et al*.	2009	Type2 DM	64.0/62.4	48/49	Pioglitazone, 30 mg/d	6
Kaneda *et al*.	2009	DM and non-DM	67.0/67.0	48/48	Pioglitazone, 15–30 mg/d	6
Hong *et al*.	2010	Type2 DM	63.5/62.4	47/47	Pioglitazone, 30 mg/d	8
García-García *et al*.	2012	Type2 DM	62.4/60.4	113/118	Rosiglitazone,4–8 mg/d	18
Lee *et al*.	2013	Type2 DM	60.3/61.9	60/61	Pioglitazone, 15 mg/d	12

TZDs, thiazolidinediones; Cont, control; DM, diabetes mellitus.

**Figure 2:**
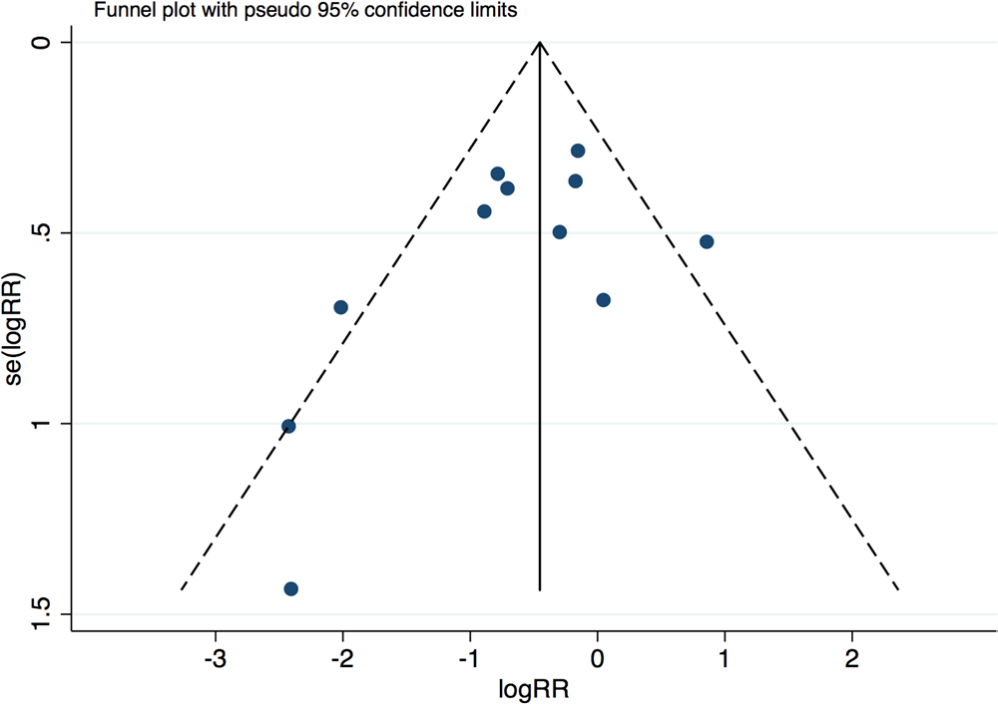
Funnel plot for the studies included based on the events of ISR.

All 14 RCTs included for pooling analysis had high qualities and relatively low risks of bias according to the Cochrane Collaboration tool. No significant publication bias was found by a funnel plot (Fig. 2) or revealed by the Egger’s and Begg’s tests based on the outcome of ISR (Egger’s: p = 0.163, Begg’s: p = 0.161).

### Primary End Points

**Figure 3:**
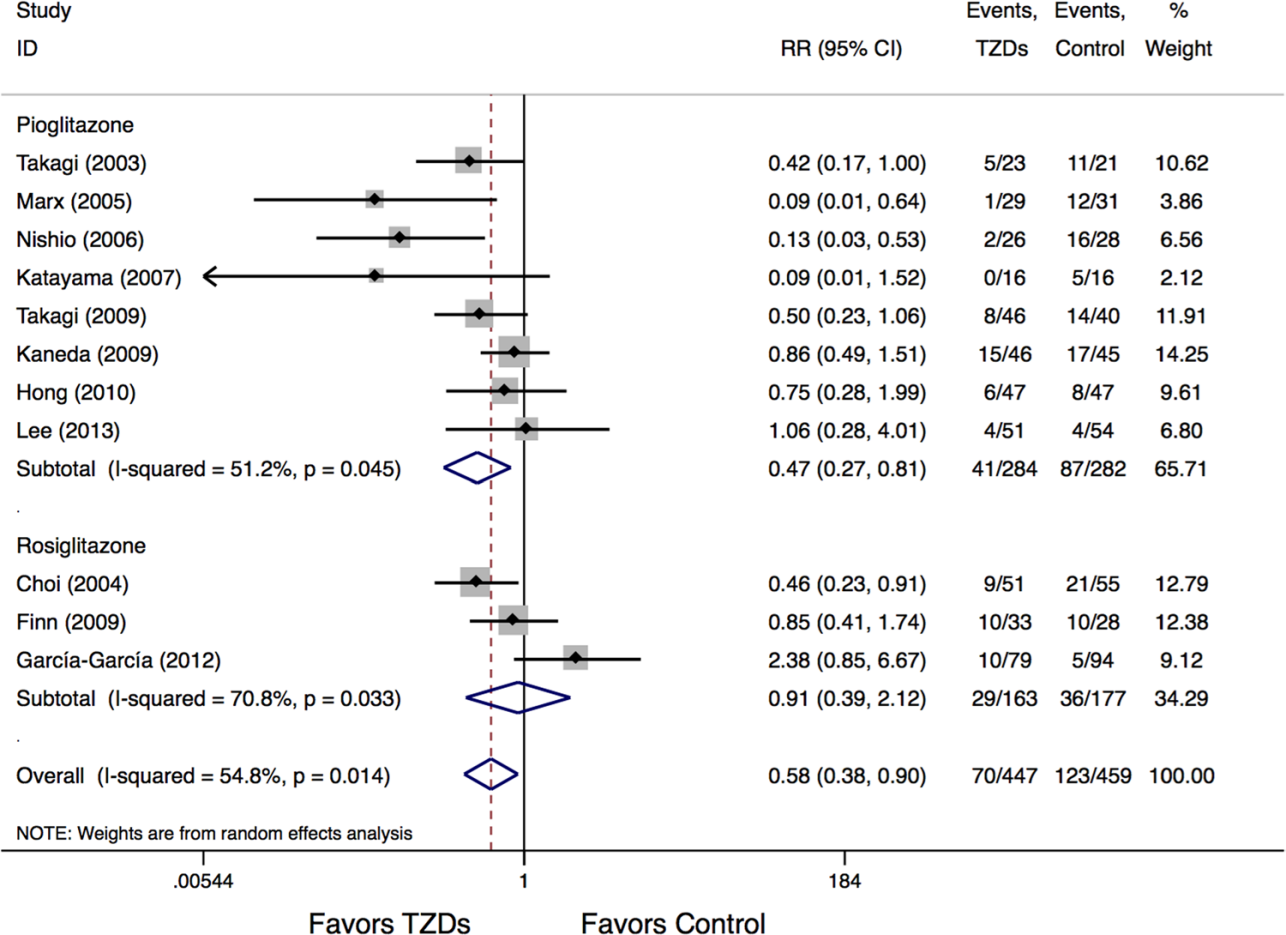
Meta-Analysis for ISR events.

The ISR rate was 15.7% in the TZDs group compared with 26.8% in the control group (RR:0.58, 95% CI:0.38 to 0.90, p = 0.016) (Fig. 3). However, moderate heterogeneity for this analysis was detected (I^2^ = 54.8%) and was further addressed by subgroup analysis according to the different TZDs type used. The results showed that the ISR was 14.4% in studies treated with pioglitazone and 30.9% in the control group (RR:0.47, 95% CI:0.27 to 0.81, p = 0.006), whereas analysis of studies treated with rosiglitazone showed an ISR rate of 17.8% and 20.3% in rosiglitazone and the control group, respectively (RR:0.91, 95% CI:0.39 to 2.12, p = 0.823) (Fig. 3). Heterogeneity was still observed for both subgroups.

**Figure 4:**
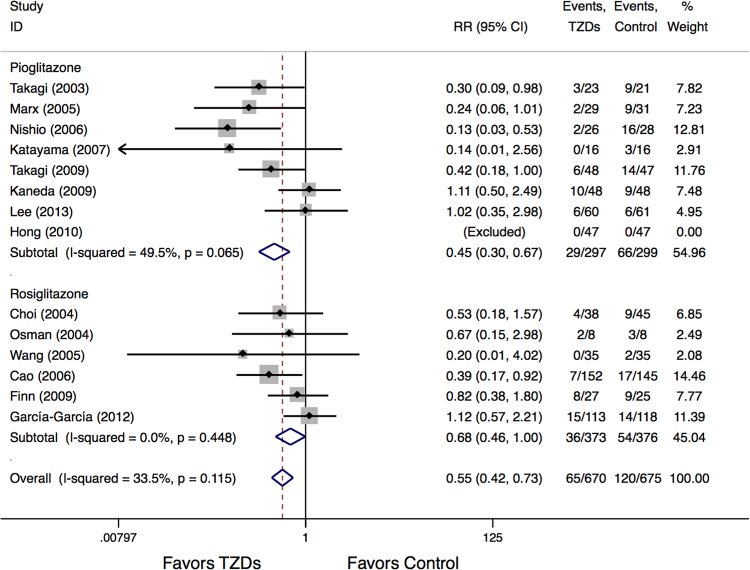
Meta-Analysis for TLR events.

TLR events occurred in 9.7% patients treated with TZDs compared to 17.8% of patients in the control group. TZDs treatment was associated with a significant reduction in TLR events (RR:0.55, 95%CI 0.42 to 0.73, P < 0.05) (Fig. 4). Additionally, in the subgroup analysis, both pioglitazone and rosiglitazone treatment resulted in significant reduction in TLR (RR:0.45, P < 0.05, RR:0.68, P < 0.05, respectively). No significant heterogeneity was found both for overall analysis (I^2^ = 33.5%) and subgroup analysis according to the TZDs type used (I^2^ = 49.5% for pioglitazone and I^2^ = 0% for rosiglitazone) (Fig. 4).

### Secondary End Points

**Figure 5:**
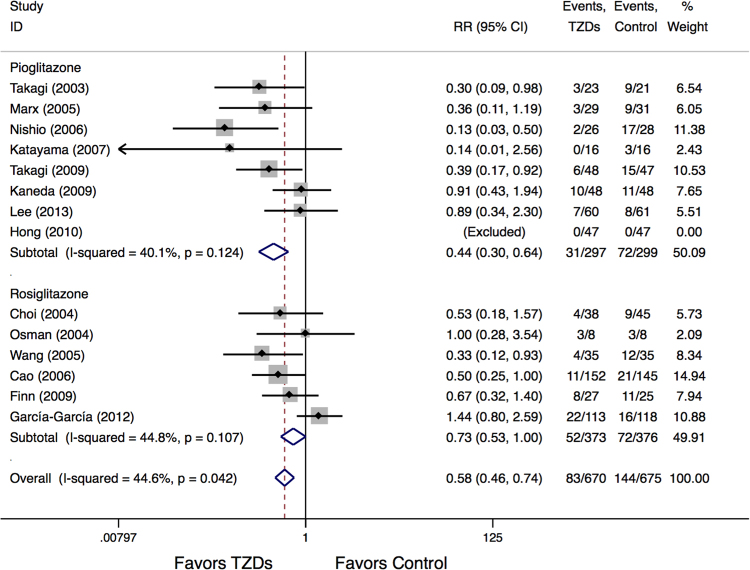
Meta-Analysis for MACE events.

No significant heterogeneity was detected for the analysis of the incidence of MACE across the studies (I^2^ = 44.6%). The results demonstrated that the treatment with TZDs was associated with a significant reduction of MACE (RR:0.58, 95% CI:0.46 to 0.74, P < 0.05) (Fig. 5). Further subgroup analysis showed that pioglitazone treatment resulted in significant MACE reduction (RR:0.44, P < 0.05), whereas no significant association was observed between rosiglitazone treatment and MACE (RR:0.73, P = 0.053) (Fig. 5).

Comparison of other QCA results, including late lumen loss, minimum lumen diameter and percentage stenosis during follow-up, are exhibited in Table 2. Results demonstrated that treatment with TZDs resulted in less late lumen loss (SMD: −0.42, P < 0.05), greater minimum lumen diameter (SMD:0.24, P < 0.05) and lower percentage stenosis (SMD: −0.39, P < 0.05). The heterogeneity was large for all three analysis (I^2^ > 50%); thus, further subgroup analysis was performed. Heterogeneity remained moderate and results showed that pioglitazone treatment exhibited significant influence on LLL, MLD and PS (P < 0.05 for all), whereas no relationship between rosiglitazone treatment and these three targets was determined(p > 0.05 for all).

**Table 2:** Comparison of the results of angiographic data.

Study (year)	Late Lumen Loss [mean(SD)]		Minimum Lumen Diameter [mean(SD)]		Percentage Stenosis [mean(SD)]	
	TZDs	Control	TZDs	Control	TZDs	Control
Takagai *et al*. (2003)	NR	NR	2.00(0.50)	1.50(0.60)	32.0(16.0)	47.0(16.0)
Choi *et al*. (2004)	0.65(0.73)	1.20(0.97)	2.49(0.88)	1.91(1.05)	23.0(23.4)	40.6(31.9)
Osman *et al*. (2004)	NR	NR	1.50(1.10)	1.40(0.90)	55.4(26.1)	57.7(28.3)
Marx *et al*. (2005)	0.88(0.41)	1.08(0.85)	2.14(0.46)	1.94(0.91)	22.1(12.7)	33.3(23.3)
Wang *et al*. (2005)	NR	NR	NR	NR	NR	NR
Cao *et al*. (2006)	NR	NR	NR	NR	NR	NR
Nishio *et al*. (2006)	0.30(0.66)	1.43(1.04)	NR	NR	NR	NR
Katayama *et al*. (2007)	0.56(0.38)	0.97(0.46)	NR	NR	21.5(9.44)	38.6(17.4)
Finn *et al*. (2009)	0.62(0.59)	0.70(0.67)	1.61(0.57)	1.60(0.78)	NR	NR
Takagi *et al*. (2009)	0.69(0.52)	1.00(0.49)	1.83(0.56)	1.57(0.65)	26.2(16.6)	36.0(23.1)
Kaneda *et al*. (2009)	0.92(0.87)	1.27(0.73)	1.66(0.79)	1.53(0.75)	42.0(28.0)	48.0(25.0)
Hong *et al*. (2010)	0.41(0.40)	0.65(0.54)	2.30(0.41)	2.09(0.53)	20.0(14.0)	28.0(17.0)
García-García *et al*. (2012)	0.87(0.58)	0.79(0.34)	1.71(0.51)	1.74(0.51)	35.9(19.0)	32.7(13.2)
Lee *et al*. (2013)	0.35(0.57)	0.31(0.60)	2.25(0.55)	2.35(0.59)	20.0(13.5)	18.5(16.8)
SMD (95% CI)						
For pioglitazone studies	−0.53(−0.84 to −0.22)*		0.30(0.03 to 0.57)*		−0.48(−0.78 to −0.18)*	
For rosiglitazone studies	−0.17(−0.67 to 0.32)		0.15(−0.19 to 0.49)		−0.16(−0.78 to 0.46)	
Overall	−0.42(−0.69 to −0.14)*		0.24(0.04 to 0.44)*		−0.39(−0.67 to −0.12)*	

^*^p < 0.05, TZDs,thiazolidinediones; SD, standard deviation; SMD, standard mean difference; NR, not reported.

IVUS data were provided in eight studies^19,21–24,26–28^, and the results demonstrated that TZDs treatment was associated with significant reduction in neointimal area (SMD: −0.552, 95%CI −0.853 to −0.250, P < 0.05) and neointimal index (SMD: −0.550, 95%CI −0.990 to −0.111, P < 0.05). Moderate to large heterogeneity was detected for both analysis. Further subgroup analysis exhibited significantly lower neointimal area (SMD: −0.585, 95%CI −0.910 to −0.261, P < 0.05) and neointimal index (SMD: −0.704, 95%CI −1.071 to −0.337, P < 0.05) in pioglitazone-treated patients, while no significant influences on both outcomes were observed in rosiglitazone-treated patients((p > 0.05 for both).

## Discussion

Results indicated a significant clinical benefit for patients after stent implantation with the addition treatment of TZDs in reducing events of ISR, TLR and MACE. The results of the QCA examinations, including LLL, MLD and PS, and IVUS results, including neointimal area and neointimal index during follow-up also further support this conclusion.

The precise mechanisms of restenosis have not been thoroughly elucidated to date. The development of intimal hyperplasia after stent-implantation induced by vascular injury and inflammation response plays a crucial role in the progression of ISR.

As insulin-sensitizing agents, TZDs have been used widely for diabetes patients and have been demonstrated to have the effects of both anti-inflammation and anti-proliferation mediated by binding to PPAR-γ^30–33^ and eventually attenuate the development of intimal hyperplasia after PCI to reduce the rates of ISR and TLR. The significant reduced neointimal area and neointimal index in TZDs-treated patients from IVUS procedure further confirmed these effects, which may also be independent of glycaemic and lipid control^34^.

Results showed that both rosiglitazone and pioglitazone treatments led to a significant reduction in the events of TLR, which was in consistent with results reported by previous studies^35^. However, there were significant differences between rosiglitazone and pioglitazone treatment as for ISR and MACE events; the QCA results including LLL, MLD and PS; and the IVUS results. Pioglitazone showed significant benefits whereas no significant relationship was detected between rosiglitazone and all those results.

The difference may be partly explained by the different gene modulation patterns and biological effects^36^ between the two agents. According to several studies, pioglitazone increases low-density lipoprotein (LDL), high-density lipoprotein (HDL) and triglycerides (TG) levels, whereas rosiglitazone mainly affects LDL^37,38^. Pioglitazone also has properties of stabilizing plaque^39^, enhancing apoptosis^40^ and suppressing fibrin formation^41^, which might contribute to its cardiovascular benefits.

However, previous studies showed that PPAR-γ could prevent arteriosclerosis through its anti-inflammatory effects^42^; in-stent restenosis was also demonstrated to be associated with insulin resistance but not lipids^34^.

The fact that few studies investigated rosiglitazone to investigate its effects beyond anti-diabetes after its restrictions were imposed may also partly contribute to the difference. It is interesting to see the imbalances between the cardiovascular effects of rosiglitazone and pioglitazone, and further studies are warranted to make this difference clear and definite.

The present analysis demonstrated that TZDs use was associated with a significant reduction of MACE events, especially for pioglitazone treatment. It should be noted that, two previous published meta-analyses indicated that rosiglitazone treatment resulted in a significant increased risk of myocardial infarction^13,43^, leading to the imposition of strict restrictions on its clinical use by the U.S. Food and Drug Administration (FDA) and the China Food and Drug Administration (CFDA). Conversely, pioglitazone has been shown in many studies to be associated with decreased risk of mortality and myocardial infarction^44,45^.

Reevaluation of the RECORD trial data in 2013 revealed that rosiglitazone did not associated with significant negative cardiovascular outcomes^14^, and its clinical restrictions were removed. Ten-year results of the PROactive trial showed that pioglitazone failed to significantly reduce cardiovascular events^46^. In addition, no relationship was found between rosiglitazone and MACE events in the present studies, and favourable benefits were even seen for rosiglitazone.

According to the evidences available, TZDs treatment, including rosiglitazone and pioglitazone, has significant benefits for patients after PCI without remarkably increased cardiovascular risks. The results should still be interpreted with caution due to the moderate heterogeneity and the inconsistency among studies.

### Study limitations

The present meta-analysis was performed based on 14 high-quality RCTs with 1350 patients; however, several limitations should still be noted. First, potential publication biases were inevitable to a certain extent, as considerable heterogeneities were detected for ISR, the QCA and IVUS results. Only half of the included studies provided IVUS results and IVUS procedure was not performed for every patient as routine in each study; thus, it may could not represent overall patients included in this study. Second, as only subgroup analysis of different TZDs type was performed, the lack of subgroup analysis, including stents type implanted and the dosage of TZDs, might contribute to the bias for this study. Finally, the follow-up lengths were abbreviated, as the longest was 18 months, which may be insufficient to measure the rates of ISR, TLR and MACE. The findings in our study might be lack of sufficient power and should be interpreted with caution. Further large-scale RCTs are needed to confirm the findings of this study.

## Conclusions

TZDs treatment for patients after PCI is associated with significant reduction in ISR, TLR and MACE. Subgroup analysis demonstrated that pioglitazone treatment showed more benefits than rosiglitazone. No significantly increased cardiovascular risk was detected for TZDs, especially for rosiglitazone. More large-scale RCTs are warranted to confirm these results further.
